# 3,4-*O*-Isopropyl­idene-2-*C*-methyl-d-galactonolactone

**DOI:** 10.1107/S1600536810001613

**Published:** 2010-01-20

**Authors:** N. Dai, S. F. Jenkinson, G. W. J. Fleet, D. J. Watkin

**Affiliations:** a Department of Organic Chemistry, Chemistry Research Laboratory, University of Oxford, Mansfield Road, Oxford OX1 3TA, England; b Department of Chemical Crystallography, Chemistry Research Laboratory, University of Oxford, Mansfield Road, Oxford OX1 3TA, England

## Abstract

X-ray crystallography unequivocally confirmed the stereochemistry of the 2-*C*-methyl group in the title mol­ecule, C_10_H_16_O_6_, in which the 1,5-lactone ring exists in a boat conformation. The use of d-galactose in the synthesis determined the absolute stereochemistry. The crystal exists as O—H⋯O hydrogen-bonded layers in the *ab* plane, with each mol­ecule acting as a donor and acceptor for two hydrogen bonds.

## Related literature

For related literature on branched sugars, see: Booth *et al.* (2008 ▶, 2009 ▶); da Cruz *et al.* (2008 ▶); Hotchkiss *et al.* (2006 ▶, 2007 ▶); Jenkinson *et al.* (2007 ▶); Jones *et al.* (2007 ▶, 2008 ▶); Rao *et al.* (2008 ▶). For the conformations of related 1,5-lactones, see: Baird *et al.* (1987 ▶); Booth *et al.* (2007*a* ▶,*b* ▶); Bruce *et al.* (1990 ▶); Punzo *et al.* (2005 ▶, 2006 ▶).
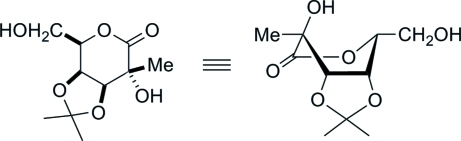


## Experimental

### 

#### Crystal data

C_10_H_16_O_6_
*M* *_r_* = 232.23
Monoclinic, 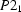

*a* = 6.0553 (2) Å
*b* = 11.3612 (4) Å
*c* = 8.2946 (3) Å
β = 105.0854 (14)°
*V* = 550.97 (3) Å^3^
*Z* = 2
Mo *K*α radiation
μ = 0.12 mm^−1^
*T* = 150 K
0.50 × 0.40 × 0.10 mm

#### Data collection

Nonius KappaCCD diffractometer
Absorption correction: multi-scan (*DENZO*/*SCALEPACK*; Otwinowski & Minor, 1997 ▶) *T*_min_ = 0.91, *T*_max_ = 0.99
5558 measured reflections
1314 independent reflections
1229 reflections with *I* > 2σ(*I*)
*R*_int_ = 0.028

#### Refinement

*R*[*F* ^2^ > 2σ(*F* ^2^)] = 0.029
*wR*(*F* ^2^) = 0.068
*S* = 0.98
1313 reflections
145 parameters
1 restraint
H-atom parameters constrained
Δρ_max_ = 0.22 e Å^−3^
Δρ_min_ = −0.18 e Å^−3^

### 

Data collection: *COLLECT* (Nonius, 2001 ▶); cell refinement: *DENZO*/*SCALEPACK* (Otwinowski & Minor, 1997 ▶); data reduction: *DENZO*/*SCALEPACK*; program(s) used to solve structure: *SIR92* (Altomare *et al.*, 1994 ▶); program(s) used to refine structure: *CRYSTALS* (Betteridge *et al.*, 2003 ▶); molecular graphics: *CAMERON* (Watkin *et al.*, 1996 ▶); software used to prepare material for publication: *CRYSTALS*.

## Supplementary Material

Crystal structure: contains datablocks global, I. DOI: 10.1107/S1600536810001613/lh2976sup1.cif

Structure factors: contains datablocks I. DOI: 10.1107/S1600536810001613/lh2976Isup2.hkl

Additional supplementary materials:  crystallographic information; 3D view; checkCIF report

## Figures and Tables

**Table 1:** Hydrogen-bond geometry (Å, °)

*D*—H⋯*A*	*D*—H	H⋯*A*	*D*⋯*A*	*D*—H⋯*A*
O8—H81⋯O1^i^	0.81	1.99	2.771 (3)	162
O1—H11⋯O6^ii^	0.86	1.99	2.737 (3)	145

Symmetry codes: (i) 
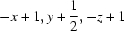
; (ii) 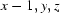.

**Fig. 1.**
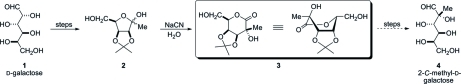
Synthetic Scheme

**Fig. 2.**
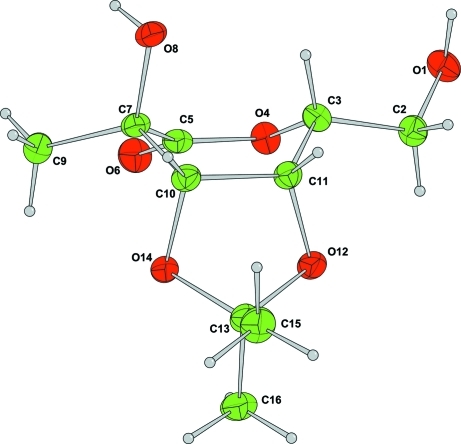
The title compound with displacement ellipsoids drawn at the 50% probability level. H atoms are shown as spheres of arbitary radius.

**Fig. 3.**
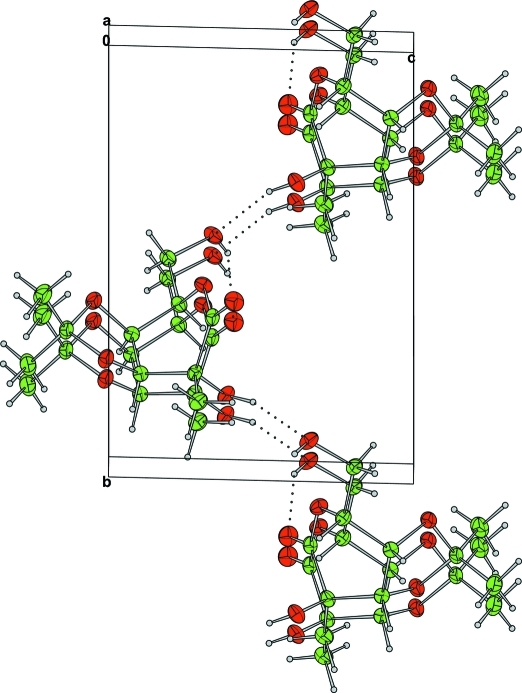
Packing diagram of the title compound projected along the a-axis.Hydrogen bonds are shown by dotted lines.

**Fig. 4.**
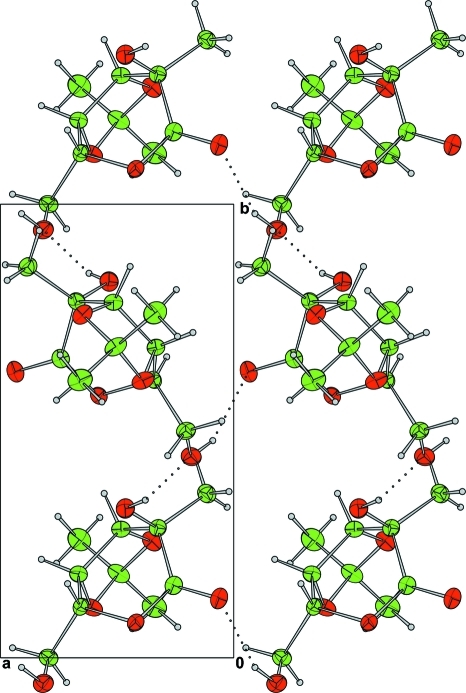
Packing diagram of the title compound projected along the c-axis.Hydrogen bonds are shown by dotted lines.

## supplementary crystallographic information

### Comment

2-*C*-Methyl branched sugars constitute a class of rare sugars with chemotherapeutic potential (Rao *et al.*, 2008; Jones *et al.*, 2008; Booth *et al.*, 2008) as well as being chirons for the enantiospecfic synthesis of complex targets (Hotchkiss *et al.*, 2006; Hotchkiss *et al.*, 2007; da Cruz *et al.*, 2008; Booth *et al.*, 2009) including 2'-*C*-methyl nucleosides (Jenkinson *et al.*, 2007). In a project to investigate the physical and biological properties of 2-*C*-methyl-D-galactose 4, D-galactose 1 [the use of which determines the absolute stereochemistry of the product] was converted by a number of steps to the lactols 2 (Fig. 1) (Jones *et al.*, 2007). The reaction of 2 with sodium cyanide in water gave a chain extension to afford a single isolated crystalline product 3 (Fig. 2). 3,4-*O*-Isopropylidene-1,5-lactones, such as 3, invariably crystallize in a boat conformation (Baird *et al.*, 1987; Bruce *et al.*, 1990; Punzo *et al.*, 2005); the diastereoselectivity may be rationalized by the formation of the galactono-lactone 3 with less steric congestion (Punzo *et al.*, 2006; Booth *et al.*, 2007*a*; Booth *et al.*, 2007*b*) than in the epimeric talono-lactone. The structure of 3 is confirmed by the X-ray crystallographic analysis reported in this paper. The lactone 3 is an intermediate for the unambiguous synthesis of 2-*C*-methyl-D-galactose 4.

The 6-membered lactone ring adopts a boat conformation with the hydroxy group rather than the methyl group in the flagpole position (Fig. 2). The title compound exists as O—H···O hydrogen bonded layers of molecules in the *ab*-plane (Fig. 3, Fig. 4). Each molecule acts as a donor and acceptor for 2 hydrogen bonds. Only classical hydrogen bonds have been considered.

### Experimental

The title compound was recrystallized by vapour diffusion from a mixture of ethyl acetate and cyclohexane: m.p. 423–429 K; [α]_D_^25^ +102.7 (*c*, 0.995 in MeOH).

### Refinement

In the absence of significant anomalous scattering, Friedel pairs were merged and the absolute configuration was assigned from the starting material.

One outlying reflection was omitted for the refinement as it was thought to be partially occluded by the beam stop.

The H atoms were all located in a difference map, but those attached to carbon atoms were repositioned geometrically. The H atoms were initially refined with soft restraints on the bond lengths and angles to regularize their geometry (C—H in the range 0.93–0.98, O—H = 0.82 Å) and *U*_iso_(H) (in the range 1.2–1.5 times *U*_eq_ of the parent atom), after which the positions were refined with riding constraints.

### Crystal data

**Table:**

C_10_H_16_O_6_	*F*(000) = 248
*M**_r_* = 232.23	*D*_x_ = 1.400 Mg m^−^^3^
Monoclinic, *P*2_1_	Mo *K*α radiation, λ = 0.71073 Å
Hall symbol: P 2yb	Cell parameters from 1236 reflections
*a* = 6.0553 (2) Å	θ = 5–27°
*b* = 11.3612 (4) Å	µ = 0.12 mm^−^^1^
*c* = 8.2946 (3) Å	*T* = 150 K
β = 105.0854 (14)°	Plate, colourless
*V* = 550.97 (3) Å^3^	0.50 × 0.40 × 0.10 mm
*Z* = 2	

### Data collection

**Table:**

Nonius KappaCCD diffractometer	1229 reflections with *I* > 2σ(*I*)
graphite	*R*_int_ = 0.028
ω scans	θ_max_ = 27.5°, θ_min_ = 5.2°
Absorption correction: multi-scan (*DENZO*/*SCALEPACK*; Otwinowski & Minor, 1997)	*h* = −7→7
*T*_min_ = 0.91, *T*_max_ = 0.99	*k* = −14→14
5558 measured reflections	*l* = −10→10
1314 independent reflections	

### Refinement

**Table:**

Refinement on *F*^2^	Primary atom site location: structure-invariant direct methods
Least-squares matrix: full	Hydrogen site location: inferred from neighbouring sites
*R*[*F*^2^ > 2σ(*F*^2^)] = 0.029	H-atom parameters constrained
*wR*(*F*^2^) = 0.068	Method = Modified Sheldrick *w* = 1/[σ^2^(*F*^2^) + (0.03*P*)^2^ + 0.19*P*], where *P* = [max(*F*_o_^2^,0) + 2*F*_c_^2^]/3
*S* = 0.98	(Δ/σ)_max_ = 0.0002
1313 reflections	Δρ_max_ = 0.22 e Å^−^^3^
145 parameters	Δρ_min_ = −0.18 e Å^−^^3^
1 restraint	

### Fractional atomic coordinates and isotropic or equivalent isotropic displacement parameters (Å^2^)

**Table:**

	*x*	*y*	*z*	*U*_iso_*/*U*_eq_	
O1	0.1833 (2)	0.44365 (15)	0.34373 (17)	0.0239	
C2	0.2057 (3)	0.50024 (18)	0.1948 (2)	0.0213	
C3	0.3440 (3)	0.61260 (18)	0.2310 (2)	0.0182	
O4	0.5789 (2)	0.57762 (14)	0.31079 (16)	0.0207	
C5	0.7424 (3)	0.65953 (18)	0.3392 (2)	0.0190	
O6	0.9366 (2)	0.63080 (16)	0.41067 (17)	0.0258	
C7	0.6739 (3)	0.78590 (18)	0.2822 (2)	0.0185	
O8	0.5348 (2)	0.82928 (15)	0.38453 (17)	0.0224	
C9	0.8833 (3)	0.86206 (19)	0.2930 (3)	0.0236	
C10	0.5160 (3)	0.78355 (18)	0.1047 (2)	0.0193	
C11	0.3342 (3)	0.68457 (18)	0.0755 (2)	0.0190	
O12	0.3897 (2)	0.61111 (14)	−0.04874 (16)	0.0232	
C13	0.5062 (3)	0.6857 (2)	−0.1390 (2)	0.0225	
O14	0.6490 (2)	0.75665 (14)	−0.00941 (15)	0.0220	
C15	0.3374 (4)	0.7604 (2)	−0.2640 (2)	0.0300	
C16	0.6590 (4)	0.6125 (2)	−0.2164 (3)	0.0307	
H21	0.0514	0.5221	0.1280	0.0253*	
H22	0.2831	0.4457	0.1327	0.0254*	
H31	0.2876	0.6635	0.3096	0.0192*	
H91	0.8339	0.9420	0.2601	0.0333*	
H93	0.9791	0.8665	0.4043	0.0339*	
H92	0.9711	0.8323	0.2182	0.0336*	
H101	0.4445	0.8626	0.0784	0.0218*	
H111	0.1751	0.7172	0.0354	0.0205*	
H152	0.4259	0.8092	−0.3237	0.0421*	
H151	0.2464	0.8116	−0.2113	0.0424*	
H153	0.2412	0.7087	−0.3440	0.0423*	
H161	0.7445	0.6676	−0.2693	0.0459*	
H163	0.7596	0.5680	−0.1332	0.0464*	
H162	0.5654	0.5645	−0.3003	0.0462*	
H81	0.6206	0.8478	0.4729	0.0319*	
H11	0.0902	0.4790	0.3908	0.0358*	

### Atomic displacement parameters (Å^2^)

**Table:**

	*U*^11^	*U*^22^	*U*^33^	*U*^12^	*U*^13^	*U*^23^
O1	0.0268 (7)	0.0221 (7)	0.0249 (7)	0.0017 (6)	0.0104 (6)	0.0072 (6)
C2	0.0241 (9)	0.0199 (9)	0.0194 (9)	−0.0019 (8)	0.0050 (7)	0.0033 (7)
C3	0.0163 (8)	0.0196 (9)	0.0185 (8)	0.0022 (7)	0.0043 (7)	0.0024 (7)
O4	0.0184 (6)	0.0193 (6)	0.0232 (7)	0.0023 (5)	0.0035 (5)	0.0035 (5)
C5	0.0203 (9)	0.0226 (10)	0.0148 (8)	0.0012 (7)	0.0063 (7)	−0.0010 (7)
O6	0.0196 (6)	0.0291 (7)	0.0272 (7)	0.0038 (6)	0.0031 (5)	0.0015 (6)
C7	0.0194 (8)	0.0196 (9)	0.0170 (8)	0.0014 (7)	0.0057 (7)	−0.0032 (7)
O8	0.0222 (6)	0.0250 (7)	0.0207 (6)	0.0006 (6)	0.0068 (5)	−0.0069 (5)
C9	0.0227 (9)	0.0232 (10)	0.0249 (10)	−0.0026 (8)	0.0063 (8)	−0.0035 (8)
C10	0.0235 (9)	0.0176 (9)	0.0171 (8)	0.0001 (8)	0.0058 (7)	0.0002 (7)
C11	0.0214 (9)	0.0181 (9)	0.0176 (8)	−0.0007 (7)	0.0051 (7)	−0.0003 (7)
O12	0.0324 (7)	0.0206 (7)	0.0185 (6)	−0.0065 (6)	0.0100 (6)	−0.0021 (5)
C13	0.0308 (10)	0.0228 (9)	0.0144 (8)	−0.0088 (8)	0.0070 (7)	−0.0017 (7)
O14	0.0252 (7)	0.0249 (7)	0.0173 (6)	−0.0066 (6)	0.0082 (5)	−0.0036 (5)
C15	0.0354 (11)	0.0336 (12)	0.0187 (9)	−0.0047 (9)	0.0028 (8)	0.0035 (8)
C16	0.0400 (11)	0.0315 (11)	0.0239 (10)	−0.0037 (9)	0.0140 (9)	−0.0059 (9)

### Geometric parameters (Å, °)

**Table:**

O1—C2	1.430 (2)	C9—H92	0.976
O1—H11	0.864	C10—C11	1.548 (3)
C2—C3	1.513 (3)	C10—O14	1.426 (2)
C2—H21	0.985	C10—H101	0.996
C2—H22	0.997	C11—O12	1.432 (2)
C3—O4	1.459 (2)	C11—H111	1.005
C3—C11	1.516 (3)	O12—C13	1.432 (2)
C3—H31	0.995	C13—O14	1.439 (2)
O4—C5	1.334 (2)	C13—C15	1.513 (3)
C5—O6	1.216 (2)	C13—C16	1.506 (3)
C5—C7	1.534 (3)	C15—H152	0.990
C7—O8	1.430 (2)	C15—H151	0.979
C7—C9	1.519 (3)	C15—H153	0.961
C7—C10	1.533 (3)	C16—H161	0.985
O8—H81	0.808	C16—H163	0.940
C9—H91	0.973	C16—H162	0.947
C9—H93	0.956		
			
C2—O1—H11	113.6	C7—C10—O14	108.81 (14)
O1—C2—C3	112.29 (15)	C11—C10—O14	103.99 (14)
O1—C2—H21	108.0	C7—C10—H101	108.8
C3—C2—H21	107.1	C11—C10—H101	111.7
O1—C2—H22	109.3	O14—C10—H101	109.7
C3—C2—H22	108.4	C10—C11—C3	112.96 (15)
H21—C2—H22	111.8	C10—C11—O12	104.22 (14)
C2—C3—O4	106.51 (15)	C3—C11—O12	109.48 (15)
C2—C3—C11	112.89 (15)	C10—C11—H111	111.4
O4—C3—C11	110.51 (14)	C3—C11—H111	107.6
C2—C3—H31	110.7	O12—C11—H111	111.2
O4—C3—H31	108.7	C11—O12—C13	105.74 (14)
C11—C3—H31	107.5	O12—C13—O14	102.88 (13)
C3—O4—C5	118.76 (15)	O12—C13—C15	110.69 (16)
O4—C5—O6	118.55 (17)	O14—C13—C15	111.39 (17)
O4—C5—C7	118.00 (15)	O12—C13—C16	109.71 (17)
O6—C5—C7	123.44 (17)	O14—C13—C16	108.15 (17)
C5—C7—O8	107.07 (14)	C15—C13—C16	113.47 (16)
C5—C7—C9	111.12 (15)	C13—O14—C10	106.42 (14)
O8—C7—C9	112.38 (15)	C13—C15—H152	107.5
C5—C7—C10	109.30 (14)	C13—C15—H151	112.7
O8—C7—C10	105.05 (14)	H152—C15—H151	109.4
C9—C7—C10	111.64 (15)	C13—C15—H153	108.1
C7—O8—H81	106.8	H152—C15—H153	107.9
C7—C9—H91	108.9	H151—C15—H153	111.2
C7—C9—H93	112.0	C13—C16—H161	106.9
H91—C9—H93	106.6	C13—C16—H163	109.6
C7—C9—H92	110.5	H161—C16—H163	110.7
H91—C9—H92	108.9	C13—C16—H162	108.3
H93—C9—H92	109.8	H161—C16—H162	108.8
C7—C10—C11	113.78 (14)	H163—C16—H162	112.3

### Hydrogen-bond geometry (Å, °)

**Table:**

*D*—H···*A*	*D*—H	H···*A*	*D*···*A*	*D*—H···*A*
C2—H22···O14^i^	1.00	2.46	3.391 (3)	155
C3—H31···O6^ii^	1.00	2.51	3.204 (3)	127
C15—H153···O6^iii^	0.96	2.52	3.454 (3)	163
O8—H81···O1^iv^	0.81	1.99	2.771 (3)	162
O1—H11···O6^ii^	0.86	1.99	2.737 (3)	145

Symmetry codes: (i) −*x*+1, *y*−1/2, −*z*; (ii) *x*−1, *y*, *z*; (iii) *x*−1, *y*, *z*−1; (iv) −*x*+1, *y*+1/2, −*z*+1.
